# Tool transfers are a form of teaching among chimpanzees

**DOI:** 10.1038/srep34783

**Published:** 2016-10-11

**Authors:** Stephanie Musgrave, David Morgan, Elizabeth Lonsdorf, Roger Mundry, Crickette Sanz

**Affiliations:** 1 Department of Anthropology, Washington University in Saint Louis, Saint Louis, USA; 2 Lester E. Fisher Center for the Study and Conservation of Apes, Lincoln Park Zoo, Chicago, USA; 3 Congo Program, Wildlife Conservation Society, Brazzaville, Republic of Congo; 4 Department of Psychology, Franklin and Marshall College, Lancaster, USA; 5 Max Planck Institute for Evolutionary Anthropology, Leipzig, Germany.

## Abstract

Teaching is a form of high-fidelity social learning that promotes human cumulative culture. Although recently documented in several nonhuman animals, teaching is rare among primates. In this study, we show that wild chimpanzees (*Pan troglodytes troglodytes)* in the Goualougo Triangle teach tool skills by providing learners with termite fishing probes. Tool donors experienced significant reductions in tool use and feeding, while tool recipients significantly increased their tool use and feeding after tool transfers. These transfers meet functional criteria for teaching: they occur in a learner’s presence, are costly to the teacher, and improve the learner’s performance. Donors also showed sophisticated cognitive strategies that effectively buffered them against potential costs. Teaching is predicted when less costly learning mechanisms are insufficient. Given that these chimpanzees manufacture sophisticated, brush-tipped fishing probes from specific raw materials, teaching in this population may relate to the complexity of these termite-gathering tasks.

Social learning facilitates the transfer of adaptive information within groups for a wide range of animal taxa and can generate group-specific behavior patterns^1^,^2^,^3^. When these behaviors persist over generations and are transmitted through social learning, they are deemed cultural^4^. High-fidelity social learning is hypothesized to distinguish human from animal cultures by promoting cumulative culture^5^,^6^,^7^; identifying what mechanisms underpin the social transmission of complex behaviors among animals is thus essential for comparative studies.

Of foremost interest is teaching. A functionalist approach identifies teaching when certain criteria are fulfilled^8^,^9^,^10^ regardless of whether there is evidence of *intent* to facilitate another’s learning^11^,^12^,^13^,^14^. The most broadly applied criteria are that the behavior (1) occurs in the presence of a naïve learner, (2) at some cost or at least no benefit to the teacher, and (3) that it facilitates learning in another individual^8^. Using these criteria, strong experimental evidence for teaching has been found for meerkats^15^, ants^16^, and pied babblers^17^. Sensitivity to learner competence^15^, or evaluation^16^, and ostensive cueing^18^ have been suggested as further criteria. Linking functional criteria to cognitive correlates of candidate teaching behaviors can improve inferences about the evolutionary origins of teaching^19^,^20^ (see Table 1).

One such candidate behavior is the transfer of tools between individuals, which has been observed among wild chimpanzees in several tool-using contexts^21^,^22^,^23^,^24^,^25^. Chimpanzee tool repertoires vary between populations, and this can be attributed in part to social learning^26^,^27^,^28^. This variation could also be associated with differences in the types of social facilitation necessary to maintain behaviors that range in complexity from simple tasks, involving only a single tool and target, to more complex tasks involving the use of tool sets^29^,^30^. For example, tool transfers have been documented during termite gathering among chimpanzees in the Goualougo Triangle, Republic of Congo^31^. There, chimpanzees are highly selective for plant species used to manufacture tools^32^ and intentionally modify herb stems to fashion brush-tipped fishing probes^33^. In addition, chimpanzees use two tool sets to gather termites from epigeal (above-ground) and subterranean nests. At epigeal nests, chimpanzees may use a perforating twig to open sealed termite exit holes on the nest surface before using an herbaceous probe to fish for termites. At subterranean nests, chimpanzees must breach underground nest chambers with a durable, woody puncturing stick before fishing^30^. Teaching is predicted to evolve when it is required to facilitate learning and when the fitness benefits accrued from a pupil’s competence outweigh the costs of teaching^10^. Given the complexity of these tool tasks, we hypothesized that tool transfers from skilled chimpanzees to less competent conspecifics constitute a form of teaching.

Using remote video footage of termite gathering, we scored behavior immediately before and after fishing probe transfers to test whether transfers impose costs on donors and confer benefits to recipients. We predicted that donors would spend proportionately less time termite gathering and exhibit reduced tool use and feeding after compared to before transfers, while recipients would spend more time using tools and exhibit increased tool use and feeding after compared to before transfers.

## Transfers of fishing probes

We identified 96 transfers of fishing probes, all of which were initiated by the recipient. A subset of transfers met the criteria for analysis (see Methods). All transfers analyzed occurred between an adult female and her offspring; the one exception occurred between a sub-adult female and her infant sister. The results represent 13 unique donors and 13 unique recipients. Recipients were immature chimpanzees, including 5 females, 4 males, and 4 youngsters of unknown sex.

## Time spent using tools to gather termites

**Figure 1:**
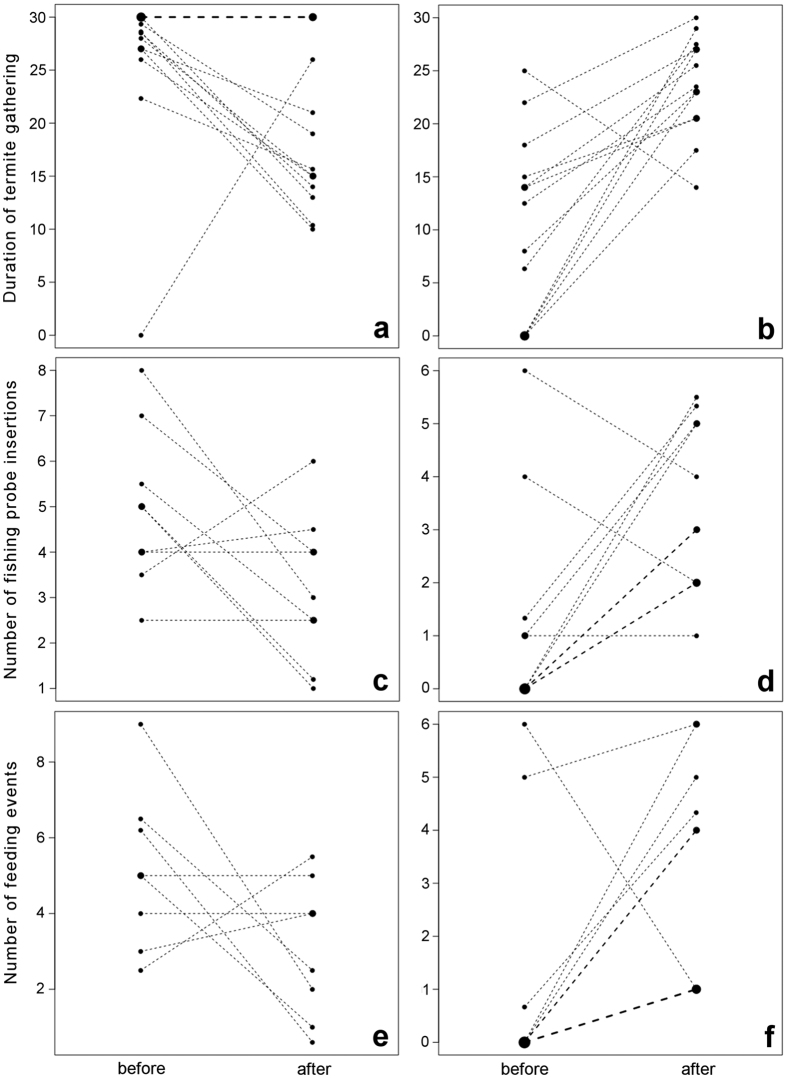
Changes in termite gathering from before to after tool transfer. The number of seconds spent using tools to gather termites *decreased* for the donor (n = 26, Fig. 1a) after relinquishing a probe to another chimpanzee (recipient, n = 24), whose time spent termite fishing *increased* (Fig. 1b). The number of fishing probe insertions also *decreased* for the donor (n = 17, Fig. 1c) and *increased* for the recipient (n = 15, Fig. 1d). Finally, the number of feeding events *decreased* for the donor (n = 15, Fig. 1e) and *increased* for the recipient (n = 14, Fig. 1f) after the transfers. Observations of the same individual or event, respectively, are denoted by a pair of points connected by a dashed line. Averages are shown for individuals with multiple observations. Tied observations (at least two individuals with the exact same value of the response) are denoted by larger points (whereby the area of the points codes the number of individuals; thicker lines have the corresponding meaning for the connections). n = number of transfers.

As shown in Fig. 1a, donors’ average time spent using tools to gather termites decreased substantially (an average of 10.6 seconds) during the 30 second interval after compared to before transfers in which donors relinquished their fishing probe to another individual (Wilcoxon signed-ranks test: *T*^+^ = 45.00, *N* = 13 donors, *P* = 0.08). Conversely, tool recipients spent on average 15 seconds more using tools to gather termites after transfer events in which they received a fishing probe (*T*^+^ = 85.5, *N* = 13 recipients, *P* = 0.003, Fig. 1b).

## Fishing probe insertions

To test whether fishing probe insertions and feeding events differed between before and after the tool transfer, we used Generalized Linear Mixed Models^34^ (GLMM), fitted separately for donors and recipients (see Extended Data Tables 1 and 2). As shown in Fig. 1c, donors (n = 9) performed significantly fewer fishing probe insertions (an average of 1.8 fewer) per 30 seconds after the transfer of a fishing probe versus prior to the transfer (GLMM: estimate ± SE = −0.49 ± 0.18, *χ*^2^ = 6.98, *df* = 1, *P* = 0.008). Of 9 donors, 5 showed an average decrease, 2 remained constant, and 2 showed increases. Those chimpanzees (n = 11) who received a tool showed a significant increase (an average of 2.8 more) in the number of probe insertions after transfers compared to their performance before (1.24 ± 0.35, *χ*^2^ = 10.44, *df* = 1, *P* = 0.001, Fig. 1d).

## Feeding events

Donors (n = 9) showed a reduction in the number of feeding events (on average 2.7 fewer) after the transfer of a fishing probe versus prior to the transfer (−0.69 ± 0.24, *χ*^2^ = 9.25, *df* = 1, *P* = 0.002; Fig. 1e). For recipients (n = 10), there was a significant increase in the average number of feeding events (an average of 2.8 more) after transfers compared to before (1.46 ± 0.43, *χ*^2^ = 8.38, *df* = 1, *P* = 0.004; Fig. 1f).

## Donor strategies buffering costs of tool transfers

Adult females occasionally transported multiple fishing probes to the termite nest in advance (n = 4 occurrences) and used one of these additional probes after a transfer. In addition, adult females deployed a second strategy (see Supplementary Video 1) in which they divided their fishing probe lengthwise and then transferred half of their tool to their offspring while retaining the other half for their own use (n = 11 occurrences). These strategies were observed in 3 and at least 6 different females, respectively. Use of a second tool or splitting of a tool lengthwise were deployed in 3 of the 6 occasions where donors’ rate of tool use or feeding increased or showed no change following a transfer. These behaviors were thus effective in buffering against the costs of tool sharing, as they enabled individuals who transferred a tool to maintain or even show an increased rate of tool use in the post-transfer period.

## Discussion

Of the functional criteria proposed to identify teaching^8^, the first is that the behavior occurs in the presence of a learner. Transfers are most common between adults and infants, principally mothers and offspring. In chimpanzees, mothers are the primary models for offspring^22^,^35^ and are most likely to benefit from offspring acquisition of tool skills. Second, teaching behaviors are predicted to be costly to the teacher. In the present study, donors incurred costs in the form of reduced time spent termite gathering, fewer fishing probe insertions, and reduced termite consumption. Third, teaching should provide the learner with increased knowledge or opportunity to acquire a skill. Tool recipients increased their time spent termite gathering, and showed higher rates of fishing probe insertions and feeding events following transfers.

Changes of tool possession from older, more competent individuals to younger, less competent individuals are distinctive in several ways from tool transfers in the opposite direction or between peers^23^, which were observed relatively rarely within this population. Active transfers in which adults move to facilitate a transfer in response to begging^23^ (see Supplementary Video 2) have only been documented when a tool changed possession from a more to a less competent individual. Further, mothers showed evidence for anticipating transfers and devising strategies that buffer associated costs, while accommodating both their offspring’s and their own need for a functional probe. Splitting a tool lengthwise is likely to be more effective for producing two viable tools than breaking the tool in half, which could result in loss of the brush tip or the tool being too short to insert to the appropriate depth. In addition, splitting a tool lengthwise or bringing a second tool in advance are both advantageous because they buffer the donor or recipient from having to locate tool material and manufacture a second tool after arrival. Tool manufacture requires identifying suitable raw material, and potentially departing from the vicinity of the nest and other conspecifics in order to do so, which increases vulnerability to predation.

An alternative interpretation of transfers, instead of teaching, is that adults relinquish tools to mitigate harassment^36^, i.e., “sharing under pressure^37^”. However, costs to donors increased rather than decreased following tool transfers, which is the opposite effect than would be predicted by the sharing under pressure hypothesis. It is the relinquishing of a tool, rather than the proximity or harassment of offspring, that is costly.

With respect to the third functional criterion, tool recipients experienced an immediate benefit through the opportunity to manipulate and use appropriate tool materials, which resulted in their increased tool use and termite consumption. Consistent with past findings that mothers did not differentially facilitate termite fishing by male and female offspring at Gombe^22^, transfers occurred to offspring of both sexes, and tool-using activity increased after transfers for 9 of the 10 recipients. Tool transfers included components both of transfer of declarative knowledge^10^, i.e., what raw material is appropriate, as well as opportunity provisioning^8^ or providing^9^ to practice termite-gathering behaviors with a suitable tool. These tools were usually transferred with the modified brush tip facing the termite nest, further scaffolding appropriate tool use. At Tai, age and skill-related shifts have been documented in mother chimpanzees’ facilitation of nut-cracking^24^. Given that the acquisition of some components of termite gathering may extend into juvenility and sub-adulthood in the Goualougo Triangle^38^, longitudinal studies will further illustrate how tool transfers impact skill acquisition as well as the extent to which tool donors are sensitive to learner competence^15^,^16^.

Teaching is hypothesized to evolve when it is optimal for transferring information that is otherwise too difficult or costly to acquire, and the limited evidence for nonhuman primate teaching comes from contexts which may fit this criterion^39^,^40^,^41^,^42^,^43^,^44^. Teaching by active facilitation of complex behaviors could be beneficial, even if the overall rate of these behaviors is low. Given that teaching may appear absent in non-experimental settings because it is difficult to measure^45^, developing rigorous methods for evaluating social learning mechanisms is necessary for comparative studies. In addition, captive research can help inform interpretation of possible cognitive correlates of teaching behaviors documented in natural settings. For example, the flexible use of coping strategies observed in this chimpanzee population indicates that donors are sensitive to and anticipate recipients’ need for a functional tool; captive experiments demonstrated that chimpanzees can attribute knowledge to others^46^ and can engage in prosocial helping under certain conditions^47^,^48^. Analyzing functional criteria alongside the potential cognitive underpinnings of social facilitation in the context of complex, learned tasks can advance our understanding of the evolution of teaching behavior across taxa and in our own lineage.

## Methods

### Subjects

Chimpanzee observations were conducted in the Goualougo Triangle, located in the southern section of the Nouabalé-Ndoki National Park (E 16°51′−16°56′; N 2°05′−3°03′), Republic of Congo. The study area encompasses 380 km^2^ of evergreen and semi-deciduous lowland forest, with altitudes ranging between 330 and 600 meter. Rainfall is bimodal, with a primary rainy season from August to November and a short rainy season in May.

### Data Collection

We placed remote video-recording devices with passive infrared sensors at termite nests to record chimpanzee visitation and tool-using behaviors^30^. Video footage was archived on hard drives and converted to MPEG for review after which we coded videos using INTERACT Version 14^50^. We screened 224 hours of footage and identified 96 fishing probe transfers, defined as the change of possession of a fishing probe from one individual to another. A subset of these transfers met criteria for inclusion in the present study. If multiple transfers occurred between the same individuals during the same visit to a termite nest, only the first transfer was included because subsequent transfers were considered nonindependent. On 4 separate visits, 2 transfers were coded in each and were included, because the transfers were separated by a minimum of approximately 10 minutes and by other intervening behaviors. Thus, we deemed each transfer event to be independent. Transfers were coded for age/sex class of donor and recipient. The resulting data set included 57 transfers of fishing probes from an older, more competent individual to an immature individual. There were two occasions in which there was a change of possession of a fishing probe between adults, four transfers from a subadult or older juvenile to an adult female, and two transfers between youngsters. These were not included in analyses due to their relative rarity.

Next we screened transfers for those in which the donor or recipient chimpanzee, or both, were continuously visible during the 30 seconds immediately before and after the transfer. We considered this time frame adequate for capturing representative behavior before and after transfers given the relatively short average duration (2.55 minutes) of termite nest visits by chimpanzees in this population^51^. In addition, because chimpanzees may go in and out of the camera field of view, coding clips continuously for the entire duration of chimpanzee presence at termite nests was not always feasible. For donors and recipients, respectively, 26 and 24 transfers allowed for determination of the proportion of time spent in active tool use. We coded behaviors including termite-gathering tool use (e.g., active insertion of fishing probes); and other behaviors such as play, inactivity, and locomotion. For a further subset of these clips, continuous visibility at a high degree of resolution for 30 seconds before and after the clips allowed for the coding of specific tool use and feeding behaviors. We further required that the donor must have initiated tool use by 30 seconds before the transfer. This was necessary in order to ensure that comparison of behavior before and after a transfer event was not systematically biased by a donor’s latency to begin termite gathering upon arrival at a termite nest. This criterion was not applied to recipients, given that immature chimpanzees often engage in a range of behaviors other than termite gathering while present at termite nests and the purpose was to discern how their behavior changed, regardless of the behavior immediately preceding the transfer. For donors, we coded fishing probe insertions and feeding events for 17 and 15 transfers, respectively; and for recipients, we coded fishing probe insertions and feeding events for 15 and 14 transfers, respectively. Fishing probe insertions involved the insertion and extraction of an herbaceous probe into an exit hole on a termite mound. Feeding elements included sweeping termites from tools, eating termites directly from the tool, or gathering termites by hand, wrist or lips from the termite nest surface.

### Analysis

In order to test whether the duration of tool use differed before and after the tool transfer we used exact^52^,^53^ Wilcoxon tests, applied separately for donors and recipients. In case individuals acted repeatedly as donor or recipient, respectively, we used the average duration per individual and time period (before or after, respectively). We did not use mixed models (see below) for the duration since it showed bottom and ceiling effects, making it impossible to find an appropriate error distribution.

For testing whether the number of feeding events and fishing probe insertions differed between before and after the tool transfer, we used Generalized Linear Mixed Models^34^ (GLMM), fitted separately for donors and recipients (see Extended Data Tables 1 and 2). These included one fixed effect denoting whether the observation was made before or after the tool transfer (“time period”). As random intercepts we included the identity of the chimpanzee and also the particular transfer event. To keep Type I error rate at the nominal level of 0.05, we included the random slope of time period within chimpanzee identity whenever we had at least two tool transfer events for at least half of the individuals^54^,^55^; this random slope was included into the models for the number of fishing probe insertions of donors and the number of feeding events of donors. We did not include the correlation between the random intercept and slope to avoid overly complex models given the small sample sizes. We used either a Poisson error structure or, in case this revealed an overdispersed response, a negative binomial error structure. Specifically, we used a Poisson model for the number of fishing probe insertions for the donor (dispersion parameter = 1.17) and negative binomial models for the other three (dispersion parameters, number fishing probe insertions, recipient: 1.48; number feeding events, donor: 1.10; number feeding events, recipient: 1.33). We tested the significance of time period using a likelihood ratio test comparing the full model with a respective null model lacking the effect^55^,^56^.

The models were fitted in R^57^ using the functions glmer or glmer.nb of the package lme4^58^ (version 1.1–10); and Wilcoxon tests were calculated using the function wilcox.exact of the package exactRankTests^59^.

## Additional Information

**How to cite this article**: Musgrave, S. *et al*. Tool transfers are a form of teaching among chimpanzees. *Sci. Rep.* **6**, 34783; doi: 10.1038/srep34783 (2016).

## Supplementary Material

Supplementary Information

Supplementary Video S1

Supplementary Video S2

## Figures and Tables

**Table 1:** Evidence for Animal Teaching.

Defining Criteria		Meerkats^15^	Ants^16^	Pied Babblers^17^	Macaques^41^,^42^	Callitrichids^43^,^44^	Felids^8^	Chimpanzees^24^,^40^
Functional	Occurs in the presence of a naïve learner^8^	**E**	**E**	*E*	E	**N, E**	**N**	**N**
	At some cost or at least no benefit to teacher^8^	**E**	**E**	*E*	E	**?**	**N**	**N**
	Facilitates learning in another individual^8^	**E**	**E**	*E*	?	**?**	**E**	**N**
	Sensitivity to learner competence^15^, evaluation^16^	**E**	**E**	*?*	?	**N, E**	**?**	**N**
	Ostensive cueing^18^	—	—	—	—	—	—	**?**
Cognitive	Ability to attribute knowledge to others^12^	—	—	—	?^49^	—	—	**N**, E 46
	Deliberate intention to facilitate learning^11^	—	—	—	—	—	—	**N**, E^47^,^48^

Included are cases where evidence for satisfaction of teaching criteria is strong in either a captive or an experimental (E) or a natural (N) setting, or present but inconclusive (?); - indicates that there is presently no evidence for a criterion. The context of teaching behavior is indicated by; bold = foraging; italics = communication, and underlined = locomotion. Plain text indicates evidence derived from studies that did not specifically assess teaching criteria. More exhaustive coverage of evidence for possible cases of animal teaching is reviewed elsewhere^8^,^9^,^10^,^19^. Chimpanzee data come from this study and the others referenced.
