# Supplementary material for: Tool transfers are a form of teaching among chimpanzees
Source: Sci Rep. 2016 Oct 11;6:34783. doi: 10.1038/srep34783 (PMC5057084; doi:10.1038/srep34783)
Supplement: Supplementary Information [file srep34783-s1.doc]

**Tool transfers are a form of teaching among chimpanzees**

Stephanie Musgrave*^1^, David Morgan^2,3^, Elizabeth Lonsdorf^4^, Roger Mundry^5^, Crickette Sanz^1,3^

^1^Department of Anthropology, Washington University

^2^Lester E. Fisher Center for the Study and Conservation of Apes, Lincoln Park Zoo

^3^Congo Program, Wildlife Conservation Society

^4^Department of Psychology, Franklin and Marshall College

^5^Max Planck Institute for Evolutionary Anthropology

***Corresponding Author:**

Stephanie L. Musgrave
Department of Anthropology
Washington University
One Brookings Drive
Campus Box 1114
Saint Louis, MO 63130
Telephone: 314-935-5252
Fax: 314-935-8535

Email: smusgrave@wustl.edu

Extended Data Table 1 | Results of GLMMs: Fixed effects

|  | estimate | SE | lower CL | upper CL | min | max |
| --- | --- | --- | --- | --- | --- | --- |
| Fishing probe insertion, donor: Intercept | 1.059 | 0.143 | 0.722 | 1.309 | 0.000 | 1.099 |
| Time period | 0.490 | 0.181 | 0.128 | 0.887 | 0.000 | 0.680 |
| Fishing probe insertion, recipient: Intercept | 1.369 | 0.197 | 0.890 | 1.699 | 0.000 | 1.421 |
| Time period | -1.244 | 0.346 | -2.032 | -0.582 | -1.609 | 0.001 |
| Feeding events, donor: Intercept | 0.981 | 0.190 | 0.627 | 1.260 | 0.802 | 1.308 |
| Time Period | 0.693 | 0.244 | 0.328 | 1.087 | 0.275 | 0.950 |
| Feeding events, recipient: Intercept | 1.273 | 0.262 | 0.571 | 1.712 | 1.146 | 1.350 |
| Time period | -1.464 | 0.433 | -2.519 | -0.620 | -1.988 | -1.227 |

Indicated are the estimated coefficients for the fixed effects together with their standard errors (SE) confidence intervals (lower CL,

upper CL) and estimations of model stability (columns headed min and max, which indicate the range of estimates derived from

excluding levels of the random effects one at a time).

Extended Data Table 2 | Results of GLMMs: Random effects

| Fishing probe insertion, donor: grp | Term | vcov | sdcor | min | max |
| --- | --- | --- | --- | --- | --- |
| Transfer | event ID | 0.000 | 0.000 | 0.000 | 0.004 |
| (chimpanzee) | Time period | 0.000 | 0.000 | 0.000 | 0.003 |
| Chimpanzee | donor ID | 0.000 | 0.000 | 0.000 | 0.234 |
| Fishing probe insertion, recipient: grp | var1 | vcov | sdcor | min | max |
| Transfer | event ID | 0.000 | 0.000 | 0.000 | 0.004 |
| Chimpanzee | recipient ID | 0.000 | 0.000 | 0.000 | 0.003 |
| Feeding events, donor: grp | Term | vcov | sdcor | min | max |
| Transfer | event ID | 0.000 | 0.000 | 0.000 | 0.001 |
| (chimpanzee) | Time period | 0.000 | 0.000 | 0.000 | 0.353 |
| Chimpanzee | donor ID | 0.000 | 0.000 | 0.000 | 0.002 |
| Feeding events, recipient: grp | Term | vcov | sdcor | min | max |
| Transfer | event ID | 0.111 | 0.333 | 0.002 | 0.688 |
| Chimpanzee | recipient ID | 0.000 | 0.000 | 0.000 | 0.636 |

Indicated are the estimated variance (vcov) and corresponding standard deviation (sdcor) for the random intercept of donor

or recipient identity and the random slope of time period within donor or recipient, respectively, together with estimations

of model stability (columns headed min and max, which indicate the range of standard deviations derived from excluding

levels of the random effects one at a time).

**Supplementary Video Clip Titles and Legends**

*Supplementary Video Clip 1.*

Title: Adult female chimpanzee divides a fishing probe lengthwise

Legend: An adult female chimpanzee at an above-ground termite nest divides her fishing probe lengthwise. She provides one half of her tool to her offspring, who uses it to successfully fish for termites, and retains the other half for her own use. This strategy produces two viable tools, which helps to buffer tool donors against the cost of transferring a tool.

*Supplementary Video Clip 2.*

Title: Adult female chimpanzee actively transfers a fishing probe

Legend: An adult female chimpanzee at an above-ground termite nest performs an active transfer of a fishing probe to her offspring, who uses it to successfully fish for termites. Active transfers involve moving to facilitate a transfer in response to begging.
